# An analysis of the association between a polymorphism of *KCNJ11* and diabetic retinopathy in a Chinese Han population

**DOI:** 10.1186/s40001-014-0075-3

**Published:** 2015-01-09

**Authors:** Nai-Jia Liu, Hui-Hui Wu, Yan-Liang Li, Zhen Yang, Xiao-Ming Tao, Yan-Ping Du, Xuan-Chun Wang, Bin Lu, Zhao-Yun Zhang, Ren-Ming Hu, Jie Wen

**Affiliations:** Department of Endocrinology and Metabolism, Huashan Hospital, Fudan University, NO. 12 Wulumuqi Mid Road, Building 0#, Jing’an District Shanghai, 200040 China; Department of Endocrinology and Metabolism, Xin Hua Hospital, Shanghai Jiao Tong University, NO. 1665 Kongjiang Road, Yangpu District Shanghai, 200020 China; Department of Endocrinology and Metabolism, Hua Dong Hospital, Fudan University, NO. 221 Yan’an West Road, Jing’an District Shanghai, 200040 China

**Keywords:** KCNJ11, Polymorphism, Diabetic retinopathy, Chinese Han population

## Abstract

**Background:**

Genome-wide association studies (GWAS) have reported that the polymorphism rs5219 of the potassium inwardly rectifying channel, subfamily J, member 11 (*KCNJ11*) is associated with type 2 diabetes mellitus (T2DM). Given that diabetic retinopathy (DR) is one of the most common microvascular complications of T2DM, GWAS have identified a number of potential susceptibility genes for DR. However, only a fraction of them have been replicated in different studies and show consistent genetic associations with the occurrence of DR. The aim of the present study was to investigate whether common variants of *KCNJ11* confer DR in a cohort of the Chinese Han population.

**Methods:**

A case–control study of 580 T2DM patients, including 105 T2DM with DR and 475 T2DM without DR was performed. A single nucleotide polymorphism (SNP) of *KCNJ11* (rs5219) was genotyped, and its association with DR was explored using a dominant genetic model. Genotyping was performed by iPLEX technology. Univariate and multivariate logistic regression (MLR) analysis controlling for confounders was conducted to evaluate the association between rs5219 and DR.

**Results:**

The A allele frequency of rs5219 was significantly higher in DR patients than that in the patients without DR (49.01% versus 38.68%, *P* <0.05). We found the minor A allele could increase the risk to develop DR (ORint = 1.58, 95% CI: 1.139 to 2.192 for allele and *P* = 0.006, ORint = 1.607, 95% CI: 1.267 to 2.038 for genotype and *P* <0.001) in the Chinese Han population.

**Conclusions:**

Our findings provided evidence that *KCNJ11* was associated with DR in Chinese Han patients with T2DM.

## Background

Type 2 diabetes mellitus (T2DM) is a multifactorial metabolic syndrome, characterized by insulin resistance and/or pancreatic β-cell dysfunction resulting from both genetic and environmental factors [1,2]. Its prevalence has increased rapidly over the last decade owing to human longevity and a surge of obesity in a lot of countries [3], which causes great economic impact on individuals, families, and health systems worldwide.

Diabetic retinopathy (DR), one of the most common microvascular complications of T2DM, is a leading cause of acquired visual loss in working-age individuals [4,5]. Blindness develops primarily from either increased DME (diabetic macular edema) or proliferation of new retinal vessels [6]. Major risk factors, such as prolonged duration diabetes, poor control of blood glucose and high blood pressure have been considered to be responsible for the onset and progression of DR [7,8]. Nevertheless, these factors do not account completely for the risk of DR. Familial aggregation and the various incidence attributed to the diverse ethnic backgrounds suggest the existence of an heritable component in DR, independent of glycemic control and duration of T2DM [9].

Recently, four small-scale GWAS with modest sample sizes conducted in Mexican-American, Chinese, and Caucasian populations indicated that several SNPs or loci had borderline or weak associations with DR in either type 1 or type 2 diabetes mellitus [10-13]. Although several candidate gene studies had reported promising genes of DR, only few of them had been replicated and showed evident genetic associations.

The potassium inwardly rectifying channel, subfamily J, member 11 (*KCNJ11*) is a pore-forming subunit of ATP-sensitive potassium (K_ATP_) channels in pancreatic β-cells [14] and plays a critical role in the regulation of insulin secretion [15]. The K_ATP_ channels consist of four subunits of the inwardly rectifying potassium channel Kir6.2 and four subunits of the sulfonylurea receptor 1 (*SUR1*), which are targets of sulfonylurea drugs. Variations of *KCNJ11* could contribute to the decreased sensitivity of the ion channel to ATP, leading to more ATP consumption, which further contributes to insulin-release impairment. E23K (rs5219) in the *KCNJ11* gene, substituting glutamate for lysine at position 23, is identified as a SNP associated with T2DM susceptibility [16]. In addition, it has been reported that *KCNJ11* is associated with the therapeutic response to sulfonylureas because of its regulation function of insulin secretion [17]. Based on the reported association between *KCNJ11* polymorphisms and T2DM, we hypothesized that this gene could also be related to the risk of DR.

Up to now, none of the risk genes of T2DM contributing to the susceptibility of DR had been reported. The aim of the present study was to investigate whether the genetic variant (rs5219) of *KCNJ11* confers DR in a Chinese Han population with T2DM.

## Methods

### Study population

Our study involved 580 Chinese patients who resided in the metropolitan area of Shanghai and had been diagnosed with T2DM. T2DM was diagnosed on the basis of the WHO criteria (1999) [18]. Known subtypes of diabetes were excluded based on antibody measurements and inheritance. Patients with diabetic ketoacidosis or ketonuria also were excluded. All the patients underwent digital non-mydriatic fundus photography, and two certified ophthalmologists diagnosed DR. Patients without DR were selected as controls. All study participants registered in the analysis were recruited from the Endocrinology and Metabolism outpatient clinics at Fudan University Huashan Hospital in Shanghai. All subjects provided written informed consent for participation in the study and donation of samples. The Ethics Committee of Huashan Hospital affiliated with Fudan University approved this protocol.

### Measurement

All participants were interviewed for the documentation of medical histories, medications, regular physical examinations, and laboratory assessment of T2DM risk factors. Physician-obtained systolic and diastolic blood pressure (BP) values were taken on the left arm of the seated participants. All participants underwent a complete hematological examination while fasting, including serum total cholesterol (TC), triglyceride (TG), blood urea nitrogen (BUN), uric acid (UA), and C-peptide (CP) levels that were measured by an enzymatic method with a chemical analyzer (Hitachi 7600–020, Tokyo, Japan). Postprandial plasma glucose (PPG) was measured 2 h after eating. Fasting plasma glucose(FPG) was measured in fasting state. The blood was centrifuged at 3,000 rpm for 10 min for plasma separation and immediately used to measure biomarkers. Serum creatinine (Cr) was measured by radioimmunoassay (Beijing Atom High-Tech Co. Ltd.). FPG and PPG were quantified by the glucose oxidase-peroxidase procedure. Glycated hemoglobin (HbA1c) was estimated by high-pressure liquid chromatography using an analyzer (HLC-723G7, Tosoh Corporation, Japan). The day-to-day and interassay coefficients of variation at the central laboratory in our hospital for all analyses were between 1% and 3%.

### Definition

Diabetes was defined as a self-reported history of physician-diagnosed T2DM or according to 1999 WHO criteria [18], which consisted of one of the following: fasting plasma glucose FPG ≥7.0 mmol/L, plasma glucose ≥11.1 mmol/L 2 hours after an oral glucose tolerance test (OGTT), or random plasma glucose ≥11.1 mmol/L. All the T2DM patients were tested for DR using digital non-mydriatic fundus photography and image analysis. Fundus photography was performed at each site following a standardized protocol. Both eyes of each participant were photographed with a 45-degree 6.3-megapixel digital non-mydriatic camera (Canon CR6-45NM, Lake Success, NY), repeated only once if necessary. DR was determined by two independent retinal specialists without knowledge of patient clinical details. The patients were classified according to the presence or absence of DR, regardless of its degree of severity. The duration was defined as the interval between the first diagnosis of diabetes and the time of enrollment in the present study. Age of onset year was the age at which an individual was diagnosed with T2DM for the first time. The clinical characteristics of participants are summarized in Table 1.

**Table 1:** **Baseline characteristics of subjects**

	**Total sample**	**With DR**	**Without DR**	***P*** **value**
Demographic information				
Number	580	475	105	-
Age (year)	64.73 ± 10.85	64.77 ± 10.96	64.54 ± 10.33	0.78
Sex (% female)	362(62.31%)	299(62.82%)	63(60%)	0.25
Height (cm)	160.39 ± 8.80	160.37 ± 8.82	160.48 ± 8.77	0.88
Weight (kg)	64.18 ± 10.64	64.25 ± 10.74	63.87 ± 10.19	0.64
SBP (mmHg)	138.76 ± 20.90	138.21 ± 21.25	141.20 ± 19.12	0.06
DBP (mmHg)	81.94 ± 11.51	81.94 ± 11.07	81.93 ± 13.31	0.99
Laboratory essays				
FPG (mmol/l)	8.69 ± 3.11	8.34 ± 2.75	10.28 ± 4.01	<0.001
CP0 (ug/l)	3.69 ± 2.13	3.71 ± 2.16	3.58 ± 1.98	0.40
PPG (mmol/l)	14.76 ± 5.71	14.01 ± 5.37	18.13 ± 5.98	<0.001
TC (mmol/l)	5.36 ± 1.11	5.35 ± 1.09	5.37 ± 1.18	0.85
TG (mmol/l)	1.97 ± 1.37	1.95 ± 1.36	2.04 ± 1.45	0.40
HbAlc (%)	7.18 ± 1.57	6.97 ± 1.40	8.12 ± 1.92	<0.001
BUN (mmol/l)	6.10 ± 1.63	6.05 ± 1.53	6.34 ± 1.99	0.02
CR (μmol/l)	67.39 ± 22.33	66.72 ± 20.05	70.43 ± 30.46	0.03
UA (mmol/l)	0.29 ± 0.08	0.29 ± 0.08	0.28 ± 0.08	0.06
Medical history				
Duration (year)	7.38 ± 6.20	7.09 ± 6.13	8.67 ± 6.39	0.001
Age of onset year	57.57 ± 10.79	57.91 ± 10.80	56.04 ± 10.62	0.02
*KCNJ11* (rs5219 A%)	231(40.51%)	181(38.68%)	50(49.01%)	0.004

BUN, blood urea nitrogen; CP0, 0 h C-peptide; Cr, creatinine; DBP, diastolic blood pressure; DR, diabetic retinopathy; FPG, fasting plasma glucose; PPG, postprandial plasma glucose; SBP, systolic blood pressure; TC, total cholesterol; TG, triglyceride; UA, uric acid.

### SNP genotyping

Peripheral venous blood samples were collected from all participants and the genomic DNA was isolated from venous blood leukocytes by the conventional phenol/chloroform method. The genetic variant (rs5219) of *KCNJ11* was genotyped using iPLEX (Sequenom, San Diego, CA, USA) with detection by the matrix-assisted laser desorption/ionization time-of-flight mass spectrometry platform. The with-DR and without-DR groups were mixed for genotyping. There was a 99.9% genotype concordance rate when duplicated samples were compared across plates.

### Statistical analysis

Continuous variables were detected by the Kolmogorov-Smirnov Test according to whether they followed normal distribution. Variables that were not normally distributed were log-transformed to approximate normal distribution for analysis. Results are described as mean ± SD or median unless stated otherwise. Differences in variables between the with-DR group and the with-DR group were determined by unpaired *t*-test. Between groups, differences in properties were accessed by *χ*^*2*^ analysis. Univariate logistic regression was performed to determine variables associated with DR and to estimate confounding factors possibly disturbing the relation of genetic variants to DR. Multivariable logistic regression (MLR) was carried out to control potential confounders for determining independent contribution of variables to DR. In order to better investigate interaction between DR and genetic variants of *KCNJ11*, we performed two analyses according to the variables of allele and genotype of *KCNJ11*, respectively. Odds ratios (OR) with 95% confidence intervals (CI) were calculated for the relative risk of genetic variants of *KCNJ11* with DR. Results were analyzed using the Statistical Package for Social Sciences for Windows version 16.0 (SPSS, Chicago, IL, USA). Tests were two-sided and a *P* value of <0.05 was considered significant.

## Results

### Clinical characteristics of subjects

**Figure 1:**
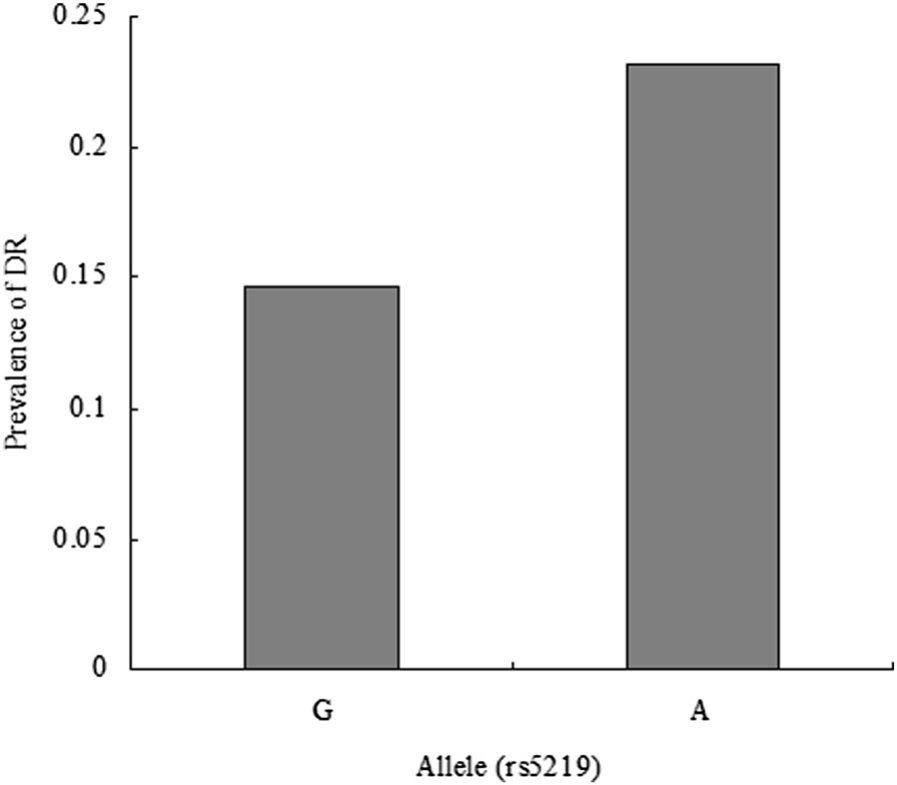
**The prevalence of diabetic retinopathy (DR) in two groups according to allele of rs5219.**

**Figure 2:**
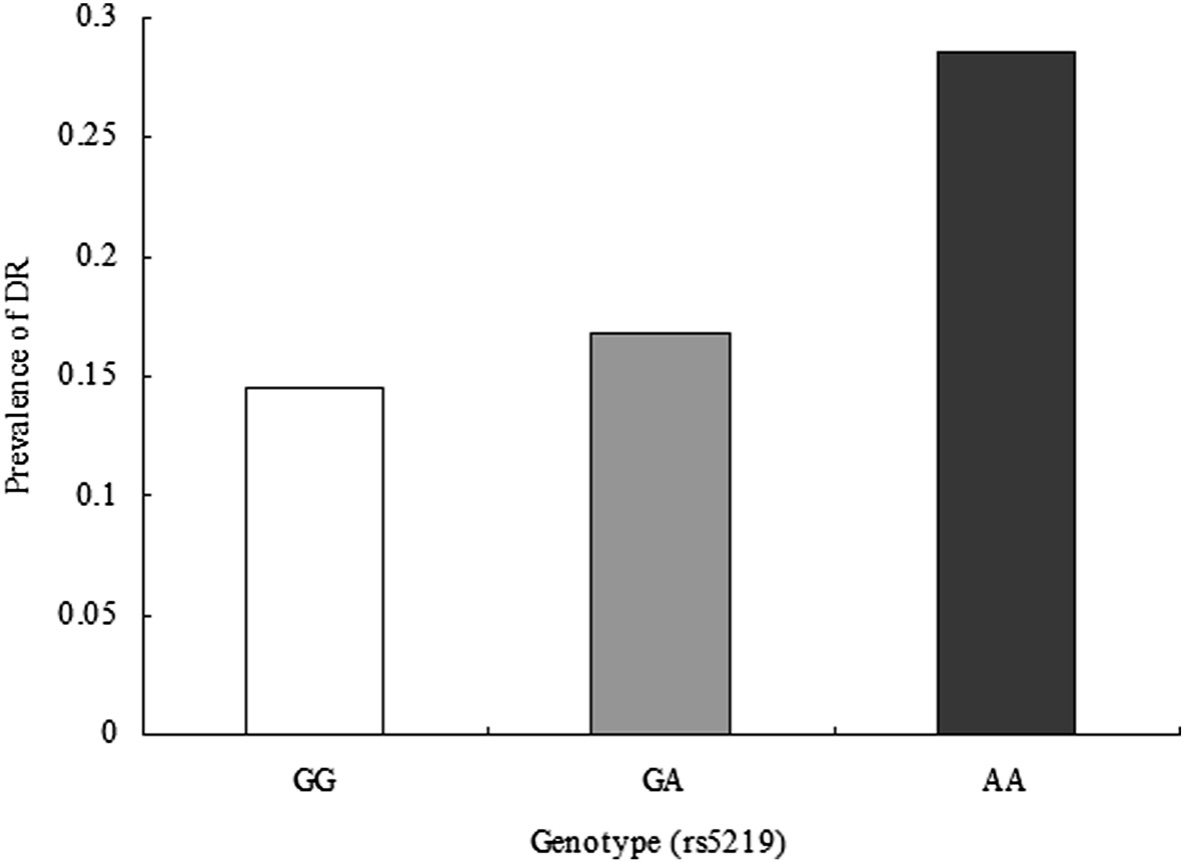
**The prevalence of diabetic retinopathy (DR) in three groups according to genotype of rs5219.**

The baseline clinical characteristics of the 580 subjects were listed in Table 1. There are 176 males and 299 females (mean age, 64.77 ± 10.9 years) in control and 42 males and 63 females (mean age, 64.54 ± 10.33 years) in case. DR patients had more weight than non-DR patients. The variables of FPG, PPG, HbAlc, BUN and Cr levels were significantly higher in cases than in controls (*P* <0.05 for all). In addition, there was a significantly longer duration of T2DM and earlier onset of T2DM in with-DR group as compared with the without-DR group (*P* <0.05 for all). Other variables of age, sex, height, weight, SBP, DBP, CP, TC, TG, UA was similar between the two groups (*P* >0.05 for all). The minor allele (A) frequency of rs5219 was 38.68% and 49.01% in controls and cases, respectively. The prevalence of DR was 14.67% and 23.21% in diabetic patients with G and A allele, respectively (Figure 1). The prevalence of DR was 14.47%, 16.85% and 28.57% in diabetic patients with GG, GA and GG genotype, respectively (Figure 2).

### Univariate logistic regression analysis for diabetes

To estimate the association of various clinical factors and DR, univariate logistic regression models were developed to include age, sex, BMI, hypertension, lipid profiles, renal function parameters and SNP (rs5219) (Table 2). The univariate logistic analyses indicated that FPG, PPG, HbAlc, BUN, Cr, duration of T2DM, onset age of T2DM were significantly associated with DR (*P* <0.05 for all). In subjects with MAF of rs5219 in *KCNJ11*, the OR for DR was 1.524 (95% CI: 1.123 to 2.069, *P* = 0.007).

**Table 2:** **Univariate analysis for risk factors of diabetic retinopathy**

**Variables**	***Β***	**S.E.**	***P value***	***OR***	**95% CI**
Age	−0.002	0.007	0.781	0.998	0.984-1.012
Sex	−0.119	0.156	0.446	0.888	0.654-1.206
BMI	−0.015	0.023	0.511	0.985	0.942-1.030
SBP	0.007	0.004	0.062	1.007	1.000-1.014
DBP	0.001	0.007	0.997	1.001	0.987-1.013
FPG	0.171	0.023	<0.001	1.186	1.135-1.240
CP0	−0.033	0.039	0.398	0.968	0.897-1.044
PPG	0.125	0.014	<0.001	1.133	1.103-1.165
TC	0.013	0.069	0.849	1.013	0.885-1.160
TG	0.044	0.052	0.402	1.045	0.943-1.158
HbAlc	0.407	0.046	<0.001	1.502	1.373-1.643
BUN	0.103	0.044	0.019	1.109	1.017-1.209
CR	0.006	0.003	0.034	1.006	1.000-1.012
UA	−2.076	1.057	0.058	0.125	0.016-1.006
Duration	0.037	0.011	0.001	1.038	1.015-1.061
Age of onset	−0.016	0.007	0.023	0.984	0.971-0.998
Family history of DM	−0.201	0.160	0.209	0.818	0.598-1.119
Alcohol	−0.842	0.444	0.058	0.431	0.181-1.028
*rs5219 A/G	0.421	0.156	0.007	1.524	1.123-2.069
*rs5219 genotype	0.432	0.112	<0.001	1.540	1.236-1.920

*KCNJ11.

BUN, blood urea nitrogen; CP0, 0 h C-peptide; Cr, creatinine; DBP, diastolic blood pressure; DR, diabetic retinopathy; FPG, fasting plasma glucose; PPG, postprandial plasma glucose; SBP, systolic blood pressure; TC, total cholesterol; TG, triglyceride; UA, uric acid.

### Multiple logistic regression analysis for diabetes

MLR demonstrated that the genetic variant (rs5219) of *KCNJ11* remained significantly different between case and control after adjustment for potential confounders (*P* = 0.006 for allele analysis and *P* <0.001 for genotype analysis, Table 3). After adjusting for confounding factors, in subjects with minor allele frequency (MAF) of rs5219 in *KCNJ11*, the OR for DR was 1.580 (95% CI: 1.139 to 2.192).

**Table 3:** **Multiple analysis for risk factors of diabetic retinopathy**

**Variables**	***Β***	**S.E.**	***P value***	***OR***	**95% CI**
*KCNJ11* (rs5219 A/G )^a^	0.458	0.167	0.006	1.580	1.139-2.192
*KCNJ11* (rs5219 genotype )^a^	0.474	0.121	<0.001	1.607	1.267-2.038

^a^adjusted for variables of Age, Sex, BMI, SBP, FPG, PPG, TC, TG, HbA1c, Duration, age of onset, Family history of DM and alcohol.

## Discussion

We conducted a case–control study to evaluate the association between *KCNJ11* (rs5219) and T2DM in a cohort of a Chinese Han population with T2DM. To our knowledge, this study is the first to find that the T2DM-susceptibility gene polymorphism (*KCNJ11* rs5219) is associated with an increased risk of DR (*P* = 0.006 for allele analysis and *P* <0.001 for genotype analysis).

T2DM is a complex and multifactorial disease resulting from a complex interaction between genes and environmental factors, whose microvascular and macrovascular complications are highly prevalent among T2DM patients. DR is one of the most common and specific microvascular complications of T2DM, which is considered to be influenced by hereditary [19,20] and environmental factors [21,22]. Given that DR is one of the leading risk factors and causes of blindness worldwide [23], the identification of risk factors for DR could lead to a decrease in vision loss associated with T2DM.

Attempting to identify risk genes of DR, numerous studies have been conducted over the past few decades [17]. Using candidate gene analysis and GWAS, investigators have identified a number of potential susceptibility genes of DR. Several biochemical pathways, including formation of advanced glycation end products (AGEs), polyol accumulation, activation of protein kinase C, oxidative stress and so on, have been proposed to mediate the pathogenesis of DR [24]. At present, three candidate genes of DR that have been extensively studied are vascular endothelial growth factor (VEGF), aldose reductase (ALR), and the receptor for advanced glycation end products (RAGE) [24,25].

Protein encoded by *KCNJ11* has a crucial function in insulin secretion, thus making it a potential susceptibility gene of T2DM. The rs5219 variant of *KCNJ11* has been widely reported to be associated with T2DM in various ethnic populations [26-28]. This gene encodes for the Kir6.2 subunit, which forms the K_ATP_ channels with the regulatory subunit sulfonylurea receptor 1 (SUR1). These channels are present in many tissues, including heart, vascular smooth muscles, and vascular endothelial cells [29] and play important roles in responses to stress [29], blood pressure regulation [30] and the physiology and pathophysiology of various tissues by coupling the metabolic state of the cells with cellular electrical activity [31].

On this basis, interestingly, a strong association is found between the common variant of *KCNJ11* (rs5219) with DR in our study (*P* = 0.006 for allele analysis and *P* <0.001 for genotype analysis), which has not been reported in previous studies. The associations between variants of *KCNJ11* and hypertension were demonstrated in Chinese [32] and Korean populations [27], suggesting a possible role of *KCNJ11* in macrovascular diseases. As DR is one of microvascular complications of T2DM, a hypothesis that *KCNJ11* may have a relationship with DR comes up. Our finding implies that *KCNJ11* (rs5219) may disturb DR susceptibility. However, the molecular mechanisms are yet to be expounded.

To date, there were no consistent and definitive genetic associations with DR in the literature, likely due to the complexity and multifactorial etiology of DR: both genetic and environmental factors play critical roles in its development and progression. Most published studies were based on small patient samples, and their case definitions were not based on the widely accepted and standardized criteria. In our study, we used the method of non-mydriatic digital stereoscopic retinal imaging (NMDSRI) for screening and diagnosing DR. NMDSRI is a sensitive and specific method for the screening and diagnosis of diabetic retinopathy, which may help improve compliance with the standards of eye care for patients with T2DM [33].

Several limitations of this study deserve comment. First, the subjects who participated in the study were recruited from Shanghai, so they may not have been representative of China as a whole. Second, we need a larger sample to further validate the effect of the polymorphism on DR risk among different ethnic populations. In addition, our study focused primary analyses on a comparison of patients with DR to patients without DR rather than comparing those with advanced or proliferative DR to those without advanced DR. The possibility of an overlap with other causes of retinopathy may have limited the proper identification of DR genes in mild stages of the disease.

## Conclusions

In summary, we identified the AA genotype or A allele of *KCNJ11* (rs5219) as a genetic risk factor for DR in T2DM patients. Further confirmatory studies on the functional effect of *KCNJ11* variants should be conducted to reveal its real contribution to T2DM.

## Abbreviations

AGEs: advanced glycation end products
ALR: aldose reductase
BUN: blood urea nitrogen
CP0: 0 h C-peptide
Cr: creatinine
DBP: diastolic blood pressure
DME: diabetic macular edema
DR: diabetic retinopathy
FPG: fasting plasma glucose
GWAS: Genome- wide association studies
HbA1c: flycated hemoglobin
MAF: minor allele frequency
MLR: multivariate logistic regression
NMDSRI: non-mydriatic digital stereoscopic retinal imaging
OGTT: oral glucose tolerance test
PPG: postprandial plasma glucose
RAGE: receptor for advanced glycation end products
SBP: systolic blood pressure
SNP: single nucleotide polymorphism
T2DM: Type 2 diabetes mellitus
SUR1: ubunit sulfonylurea receptor 1
TC: total cholesterol
TG: triglyceride
UA: uric acid
VEGF: vascular endothelial growth factor
